# Serum-free medium and hypoxic preconditioning synergistically enhance the therapeutic effects of mesenchymal stem cells on experimental renal fibrosis

**DOI:** 10.1186/s13287-021-02548-7

**Published:** 2021-08-23

**Authors:** Naoki Ishiuchi, Ayumu Nakashima, Shigehiro Doi, Ryo Kanai, Satoshi Maeda, Shinya Takahashi, Masataka Nagao, Takao Masaki

**Affiliations:** 1 Department of Nephrology, Hiroshima University Hospital, 1-2-3 Kasumi, Minami-ku, Hiroshima, Hiroshima 734-8551 Japan; 2 Center for Cause of Death Investigation Research, Graduate School of Biomedical & Health Sciences, Hiroshima University, 1-2-3 Kasumi, Minami-ku, Hiroshima, Hiroshima 734-8553 Japan; 3 Department of Forensic Medicine, Graduate School of Biomedical & Health Sciences, Hiroshima University, 1-2-3 Kasumi, Minami-ku, Hiroshima, Hiroshima 734-8553 Japan; 4 Department of Stem Cell Biology and Medicine, Graduate School of Biomedical & Health Sciences, Hiroshima University, 1-2-3 Kasumi, Minami-ku, Hiroshima, Hiroshima 734-8553 Japan; 5 TWOCELLS Company, Limited, 16-35 Hijiyama-honmachi, Minami-ku, Hiroshima, 732-0816 Japan; 6 Department of Surgery, Graduate School of Biomedical & Health Sciences, Hiroshima University, 1-2-3 Kasumi, Minami-ku, Hiroshima, Hiroshima 734-8553 Japan

**Keywords:** Mesenchymal stem cells, Hypoxic preconditioning, Serum-free conditions, Hepatocyte growth factor, Renal fibrosis

## Abstract

**Background:**

Mesenchymal stem cells (MSCs) repair injured tissue in a paracrine manner. To enhance their therapeutic properties, preconditioning with various factors has been researched. We have previously showed that MSCs cultured in serum-free medium (SF-MSCs) promote their immunosuppressive ability, thereby enhancing their anti-fibrotic effect. Here, we examined whether serum-free medium and hypoxic preconditioning synergistically enhance the therapeutic effects of MSCs on renal fibrosis in rats with ischemia–reperfusion injury (IRI).

**Methods:**

SF-MSCs were incubated under 1% O_2_ conditions (hypo-SF-MSCs) or 21% O_2_ conditions (normo-SF-MSCs) for 24 h before collection. After IRI procedure, hypo-SF-MSCs or normo-SF-MSCs were injected through the abdominal aorta. At 7 or 21 days post-injection, the rats were killed and their kidneys were collected to evaluate inflammation and fibrosis. In in vitro experiments, we investigated whether hypo-SF-MSCs enhanced secretion of anti-fibrotic humoral factors using transforming growth factor (TGF)-β1-stimulated HK-2 cells incubated with conditioned medium from hypo-SF-MSCs or normo-SF-MSCs.

**Results:**

Normo-SF-MSCs showed attenuation of senescence, which increased their proliferative capacity. Although no significant difference in cellular senescence was found between normo-SF-MSCs and hypo-SF-MSCs, hypo-SF-MSCs further increased their proliferative capacity compared with normo-SF-MSCs. Additionally, administration of hypo-SF-MSCs more strongly ameliorated renal fibrosis than that of normo-SF-MSCs. Moreover, although hypo-SF-MSCs strongly attenuated infiltration of inflammatory cells compared with the control rats, which were treated with PBS, this attenuation was almost equal between normo-SF-MSCs and hypo-SF-MSCs. In vitro experiments revealed that hypo-SF-MSCs more significantly inhibited transforming growth factor (TGF)-β/Smad signaling compared with normo-SF-MSCs. Moreover, hypoxic preconditioning increased hepatocyte growth factor (HGF) secretion even under serum-free conditions, whereas knockdown of HGF in hypo-SF-MSCs attenuated inhibition of TGF-β/Smad signaling.

**Conclusions:**

These results indicate that administration of ex vivo-expanded, hypoxia-preconditioned SF-MSCs may be a useful cell therapy to prevent renal fibrosis.

**Supplementary Information:**

The online version contains supplementary material available at 10.1186/s13287-021-02548-7.

## Background

Acute kidney injury (AKI) is common clinical problem with high mortality and morbidity [1, 2], which is often caused by loss of blood flow to the kidney during volume depletion, shock, sepsis, and renal transplantation [3, 4]. In general, most AKI patients recover their baseline renal functions, but several studies have demonstrated that a large number of AKI patients eventually develop chronic kidney disease (CKD), which suggests that AKI, even if followed by renal recovery, is a major risk factor for progression of CKD [5, 6]. Although the mechanism of AKI-to-CKD transition is incompletely understood, recent studies have reported that several pathological mechanisms, including hypoxia, microvascular rarefaction, inflammation, transforming growth factor (TGF)-β1 production, and epithelial-mesenchymal transition, are involved in AKI-to-CKD transition [7, 8]. However, there are currently few effective therapies to prevent AKI-to-CKD progression.

Mesenchymal stem cells (MSCs) are multipotent adult stem cells, which can be isolated from various tissues, such as bone marrow, adipose tissue, and umbilical cord [9, 10], and repair injured tissue via their paracrine effects [11, 12]. Several studies have demonstrated that administration of MSCs exerts beneficial effects against various renal disease models [13, 14]. However, the MSCs in these studies were cultured by the conventional method and did not exhibit sufficient therapeutic efficacy.

MSCs remain dormant under normal conditions, whereas MSCs activated by cytokines released from immune cells in damaged tissues are converted to the active form and exert anti-inflammatory effects [15, 16]. This conversion to the active form might require several days. Therefore, prior to administration, preconditioning with various factors, including cytokines [17, 18], hypoxia [19, 20], pharmacological compounds [21, 22], and genetic modification [23, 24], has been widely studied to enhance the therapeutic effects of MSCs. We have previously reported that MSCs cultured in STK (Kanto Chemical, Tokyo, Japan), which is serum-free medium containing growth factors for MSC culture, promote their immunosuppressive ability by increasing the expression of tumor necrosis factor-α-induced protein 6 (TSG-6) and inducing polarization of immunosuppressive M2 macrophages, thereby enhancing their anti-fibrotic effect [25]. Furthermore, we have recently demonstrated that hypoxia-preconditioned MSCs have enhanced immunosuppressive and anti-fibrotic abilities by increased vascular endothelial growth factor (VEGF) and hepatocyte growth factor (HGF) secretion [20]. These findings led us to the hypothesis that serum-free conditions and hypoxic preconditioning would synergistically enhance the therapeutic effects of MSCs on renal fibrosis.

In this study using human bone marrow-derived MSCs, we show that MSCs cultured in serum-free medium under 1% O_2_ conditions (hypo-SF-MSCs) potently ameliorate renal fibrosis in a rat ischemia-reperfusion injury (IRI) model. We also show that conditioned medium from hypo-SF-MSCs strongly inhibits TGF-β1-induced fibrotic changes in HK-2 cells. Additionally, we demonstrate that inhibition of HGF reduces the anti-fibrotic effect of hypo-SF-MSCs. Our data suggest that administration of hypoxia-preconditioned SF-MSCs may be a useful therapy to prevent progression of renal fibrosis.

## Methods

### Preparation of MSCs

Human MSCs were isolated from bone marrow collected from the sternum of two patients who underwent coronary artery bypass grafting. The cells were cultured in Dulbecco’s modified Eagle’s medium (DMEM; Sigma-Aldrich, St. Louis, MO, USA) with 10% FBS (Sigma-Aldrich) or STK and were designated as “10%MSCs” or “SF-MSCs”, respectively. Cells up to passage 6 were used in all experiments and counted by a TC-20 (Bio-Rad, Hercules, CA, USA). The Medical Ethics Committee of Hiroshima Graduate School of Biomedical Science permitted collection of the bone marrow (Permit number: E-1089, registered on February 2, 2018). Each patient provided written informed consent.

### Flow cytometric analysis

Flow cytometric analysis was performed in accordance with previously described methods [25]. The following antibodies were used: anti-human CD29 IgG antibody (BioLegend, San Diego, CA), anti-human CD44 IgG antibody (BioLegend), anti-human CD73 IgG antibody (BioLegend), anti-human CD90 IgG antibody (BioLegend), anti-human CD11b IgG antibody (BioLegend), anti-human CD34 IgG antibody (BioLegend), anti-human CD45 IgG antibody (BioLegend), anti-human HLA-A,B,C IgG antibody (BioLegend), and anti-human HLA-DR IgG antibody (BioLegend). The stained MSCs were analyzed by a BD FACSVerse (Becton, Dickinson and Company, Franklin Lakes, NJ). Data were assessed by FlowJo software (FlowJo, LLC; Ashland, OR).

### Differentiation experiments

To induce adipogenic or osteogenic differentiation, MSCs were cultured in adipogenic differentiation medium (Takara Bio, Shiga, Japan) or osteogenic differentiation medium (Sigma-Aldrich), respectively, for 14 days following the manufacturers’ protocols. Oil Red O (Sigma-Aldrich) and Alizarin Red S (FUJIFILM Wako Pure Chemical, Osaka, Japan) were used to analyze adipogenic and osteogenic differentiation, respectively.

### Cell proliferation assay

Proliferative activity of MSCs was analyzed by a water-soluble tetrazolium salt (WST)-1 assay (Takara Bio). MSCs (2.5 × 10^3^ cells/100 μL) were seeded in 96-well microplates and cultured in DMEM containing 10% FBS or STK under normoxic (21% O_2_) or hypoxic (1% O_2_) conditions. After incubation for 0, 12, and 24 h, 10 μL WST-1 reagent was added to each well, followed by incubation for 4 h. Absorbance was determined using a microplate reader at a wavelength of 450 nm and reference wavelength of 620 nm.

### Transwell migration assay

The migration capacity of MSCs was analyzed using a Cell Migration/Chemotaxis Assay Kit (PromoCell, Heidelberg, Germany) containing transwell chambers (24-well, 8 μm pore size). 10%MSCs and SF-MSCs (2 × 10^5^ cells/well) were seeded in upper chamber and 600 μl serum-free medium containing stromal cell-derived factor 1 (100 ng/ml, PEPROTECH, Cranbury, NJ, USA) was placed in the lower chamber. After 10 h of incubation under normoxic (21% O_2_) or hypoxic (1% O_2_) conditions, cells on the upper side of the membrane were removed with a cotton swab. Then, the upper chamber was removed and medium in the lower chamber was replaced with 550 μl of cell dissociation solution containing a dye solution. Subsequently, the upper chamber was placed in the lower chamber, followed by 1 h of incubation under normoxia (21% O_2_) and then 110 μl of the mixture in the lower chamber was transferred to a 96-well plate. A standard curve of cells that contained a dye solution was also generated in the 96-well plate following the manufacturer’s protocol. This plate was read using the fluorescence plate reader at 530/590 nm (Ex/Em). The number of migrated cells was calculated using the standard curve.

### Hypoxic preconditioning

For hypoxic preconditioning, MSCs were cultured in STK. At 70% confluence, the culture medium was replaced with fresh complete medium and the cells were incubated under 1% O_2_ conditions (hypo-SF-MSCs) or 21% O_2_ conditions (normo-SF-MSCs) for 24 h before collection. Hypoxic preconditioning was performed in a Modular Incubator Chamber (MIC 101) (Billups-Rothenberg, San Diego, CA, USA). 10%MSCs were only incubated under 21% O_2_ conditions (normo-10%MSCs).

### Animals

Male Sprague–Dawley (SD) rats (8 weeks old) were purchased from Charles River Laboratories Japan (Yokohama, Japan) and were used to induce IRI. All experimental procedures were approved by the Institutional Animal Care and Use Committee of Hiroshima University (Hiroshima, Japan) (Permit number: A16-83) and conducted in accordance with the “Guide for the Care and Use of Laboratory Animals, 8^th^ ed, 2010” (National Institutes of Health, Bethesda, MD, USA).

### Experimental animal model

SD rats were randomly divided into four groups (*n* = 5 in each group): sham, PBS, normo-SF-MSCs, and hypo-SF-MSCs groups. Renal IRI was induced by transiently clamping the unilateral renal artery. Rats were anesthetized by an intraperitoneal injection of three types of mixed anesthetic agents (medetomidine, midazolam, and butorphanol). After performing a laparotomy, the left kidney was exposed. Then, the renal pedicle was clamped using atraumatic vascular clamps for 1 h, followed by reperfusion on a heating blanket. After reperfusion, MSCs (5 × 10^5^ cells/rat) in 0.2 ml PBS were injected into the abdominal aorta clamped above and below the left renal artery bifurcation. At 7 or 21 days post-injection, the rats were killed and their left kidneys were collected to evaluate inflammation and fibrosis.

### Western blot analysis

Sample collection and western blotting were performed as described previously [20]. Rabbit polyclonal anti-Sirt1 antibody (Sigma-Aldrich), rabbit monoclonal anti-p16^INK4a^ antibody (Abcam, Cambridge, UK), mouse monoclonal anti-GAPDH antibody (Sigma-Aldrich), mouse monoclonal anti-α-SMA antibody (Sigma-Aldrich), mouse monoclonal anti-TGF-β1 antibody (Santa Cruz Biotechnology, Santa Cruz, CA, USA), rabbit monoclonal anti-phosphorylated Smad2 (p-Smad2) antibody (Cell Signaling Technology, Danvers, MA, USA), mouse monoclonal anti-Smad2 antibody (Cell Signaling Technology), mouse monoclonal anti-α-tubulin antibody (Sigma-Aldrich), rabbit monoclonal anti-CD163 antibody (Abcam), and rabbit polyclonal anti-CD68 antibody (Abcam) were used as primary antibodies. Horseradish peroxidase-conjugated goat anti-rabbit immunoglobulin G (Dako, Glostrup, Denmark) or goat anti-mouse immunoglobulin G (Dako) were used as secondary antibodies. SuperSignal West Dura or Pico system (Thermo Fisher Scientific, Rockford, IL, USA) was used to detect signals. The intensity of each band was analyzed by ImageJ software (version 1.47v; National Institutes of Health) and standardized by the level of either GAPDH or α-tubulin.

### Immunohistochemical analysis

Immunohistochemical staining of formalin-fixed, paraffin-embedded tissue Sects. (4 μm thick) was performed in accordance with previously described methods [25]. The following primary antibodies were used: mouse monoclonal anti-α-SMA antibody (Sigma-Aldrich), rabbit polyclonal anti-CD3 antibody (Dako), rabbit polyclonal anti-CD68 antibody (Abcam), and rabbit monoclonal anti-CD163 antibody (Abcam). CD3-, CD68-, and CD163-positive cells and positive areas for α-SMA staining were determined using ImageJ software by examination of five randomly selected fields (× 200) of the cortex.

### Histological analysis

Formalin-fixed, paraffin-embedded tissue Sects. (2 μm thick) were stained with hematoxylin and eosin (HE) and Masson trichrome to assess histological injury and fibrosis. The areas of interstitial fibrosis were evaluated using Lumina Vision (Mitani, Osaka, Japan) by examining five randomly selected fields (× 200) of the cortex.

### Preparation of conditioned medium

To generate conditioned medium from normo-SF-MSCs (normo-SF-MSC-CM) and hypo-SF-MSCs (hypo-SF-MSC-CM), MSCs (3 × 10^5^ cells/dish) were seeded in 10-cm dishes and cultured in DMEM containing 10% FBS or STK. At 70% confluence, the culture medium was replaced with DMEM containing 0.1% FBS, and the cells were cultured for 24 or 48 h under 1% O_2_ or 21% O_2_ conditions. Then, each medium was collected.

### Cell culture and treatments

HK-2 cells were purchased from the American Type Culture Collection (Manassas, VA, USA). The cells were cultured as described previously [25]. After serum starvation of HK-2 cells with DMEM containing 0.1% FBS or conditioned medium from MSCs for 24 h, 10 ng/ml recombinant human TGF-β1 (R&D Systems, Minneapolis, MN, USA) was added to the cells directly. After 30 min (to investigate protein levels of p-Smad2) or 24 h (to investigate protein levels of α-SMA), HK-2 cells were collected and subjected to in vitro experiments.

THP-1 cells were also obtained from the American Type Culture Collection and cultured as described previously [25]. To induce differentiation of THP-1 cells into M1 macrophages, THP-1 cells were treated with 160 nM phorbol 12-myristate 13-acetate (Sigma-Aldrich) for 48 h. Then, the medium was replaced with conditioned medium from MSCs. After 24 h, the cells were collected and subjected to in vitro experiments.

### Quantitative real-time reverse transcription-PCR

RNA extraction and real-time reverse transcription-PCR were performed in accordance with previously described methods [25]. Specific oligonucleotide primers and probes for rat TGF-β1 (assay ID: Rn00572010_m1), rat collagen type I (assay ID: Rn01463848_m1), rat collagen type III (assay ID: Rn01437681_m1), rat interleukin (IL)-1β (assay ID: Rn00580432_m1), rat IL-6 (assay ID: Rn01410330_m1), human TSG-6 (assay ID: Hs00200180_m1), and 18S rRNA (endogenous control) were obtained as TaqMan Gene Expression Assays (Applied Biosystems, Foster City, CA, USA). mRNA levels were normalized to the level of 18S rRNA.

### Enzyme-linked immunosorbent assay (ELISA)

ELISA analyses of VEGF (R&D Systems) and HGF (R&D Systems) were performed following the manufacturer’s protocols. Concentrations were normalized to the total protein content.

### Transfection of HGF siRNA

MSCs were transfected with 20 nM siRNA that targeted HGF (s6530; Applied Biosystems) or negative control siRNA (4,390,843; Applied Biosystems) using Lipofectamine 2000 Transfection Reagent (Thermo Fisher Scientific). After 24 h, the transfected cells were washed and fresh complete medium was added. At 80% confluence, these cells were used to generate conditioned medium.

### Statistical analysis

Results are expressed as means ± standard deviations (S.D.). For multiple group comparisons, one-way ANOVA followed by Bonferroni’s post-hoc test was applied. Comparisons between two groups were analyzed by the Student’s t-test. *P* < 0.05 was considered statistically significant.

## Results

### Characterization of MSCs

We first identified MSCs by morphology and flow cytometry. The cells displayed typical spindle shapes and were adherent to plastic dishes. Flow cytometry showed that the cells expressed standard MSC markers, such as CD29, CD44, CD73, CD90, and HLA-A,B,C (Additional file 1a), and did not express MSC-negative markers, such as CD11b, CD34, CD45, and HLA-DR (Additional file 1a). Additionally, we assessed the adipogenic and osteogenic differentiation abilities of MSCs. Oil Red O and Alizarin Red S staining showed that the MSCs could undergo adipogenic and osteogenic differentiations (Additional file 1b, 1c).

## Serum-free medium ameliorates senescence of MSCs

**Fig. 1:**
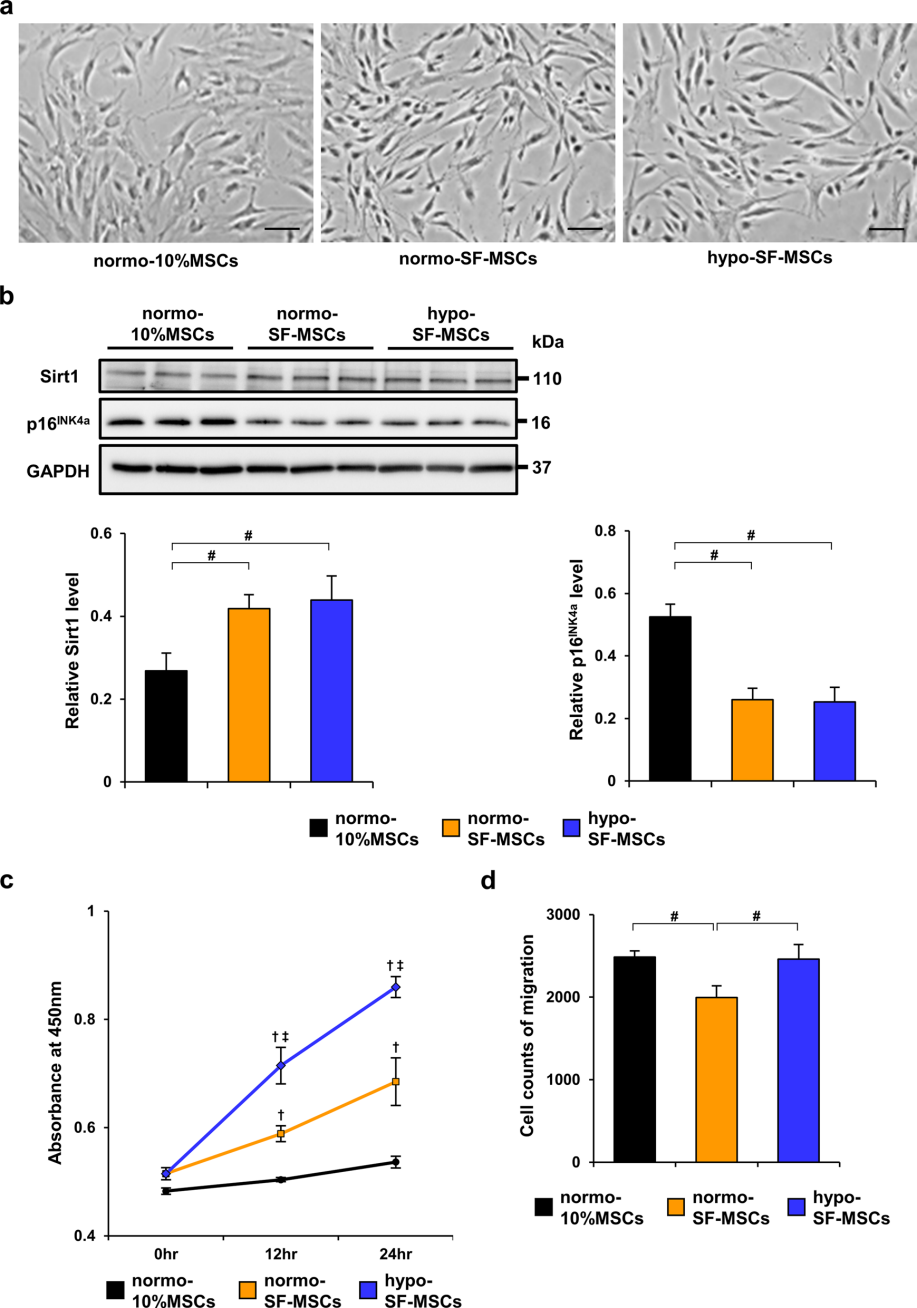
Hypo-SF-MSCs have ameliorated senescence and an enhanced proliferative ability. **a** Representative images of normo-10%MSCs, normo-SF-MSCs, and hypo-SF-MSCs at passage 8 (scale bar = 100 µm). **b** Western blot analysis of Sirt1 and p16^INK4a^ in 10%MSCs, normo-SF-MSCs, and hypo-SF-MSCs. Graphs show densitometric analyses of Sirt1 and p16^INK4a^ expression levels normalized to the GAPDH expression level (*n* = 5 in each group). **c** Surviving cells were assessed by a WST-1 assay. Graph shows the absorbance value at each time point (0, 12, and 24 h) (*n* = 5 in each group). ^†^*P* < 0.01 versus normo-10%MSCs, ^‡^*P* < 0.01 versus normo-SF-MSCs. **d** Migration activity of MSCs was analyzed using a transwell migration assay. Graph shows the numbers of migrated cells at 10 h (*n* = 4 in each group). Experiments to evaluate cell proliferation and migration were performed three times and similar results were obtained. Data are means ± S.D. ^#^*P* < 0.01 (one-way ANOVA followed by Bonferroni’s post-hoc test)

In accordance with previous studies, MSCs cultured for many passages exhibit a senescence-like morphology such as irregular shapes, enlarged cell bodies, and flattened cell bodies [26, 27]. However, unlike 10%MSCs, SF-MSCs at passage 8 did not display senescence-like morphology, but elongated spindle shapes, whereas their morphology was almost equivalent in SF-MSCs cultured under normoxia and hypoxia (Fig. 1a). Several studies have reported that downregulated expression of Sirt1 is involved in the aging process of MSCs with evidence that senescence-associated factor p16^INK4a^ is negatively regulated by Sirt1 [28, 29]. Accordingly, we next examined the expression of Sirt1 and p16^INK4a^ in MSCs. Although the protein level of Sirt1 was upregulated in SF-MSCs compared with normo-10%MSCs, there was no significant difference between normo-SF-MSCs and hypo-SF-MSCs (Fig. 1b). Conversely, expression of p16^INK4a^ was decreased in both normo-SF-MSCs and hypo-SF-MSCs compared with normo-10%MSCs, but there was no significant difference between normo-SF-MSCs and hypo-SF-MSCs (Fig. 1b).

## Hypoxic preconditioning enhances the proliferative ability and migration capacity of SF-MSCs

To assess whether the combination of serum-free medium and hypoxic preconditioning promoted the proliferative ability of MSCs, we examined the proliferative activity of normo-10%MSCs, normo-SF-MSCs, and hypo-SF-MSCs using the WST-1 assay. The absorbance value of normo-SF-MSCs was more significantly increased than that of normo-10%MSCs in a time-dependent manner and a further increase was observed in hypo-SF-MSCs (Fig. 1c), which suggested that serum-free medium and hypoxic preconditioning synergistically enhanced the proliferative ability of MSCs. Additionally, we investigated whether the combination of serum-free conditions and hypoxic preconditioning enhanced the migration capacity of MSCs by the transwell migration assay. As shown in Fig. 1d, the migration ability of normo-SF-MSCs was lower than that of normo-10%MSCs, but this reduction was canceled in hypo-SF-MSCs.

### Hypoxia-preconditioned SF-MSCs attenuate IRI-induced renal fibrosis in rats

**Fig. 2:**
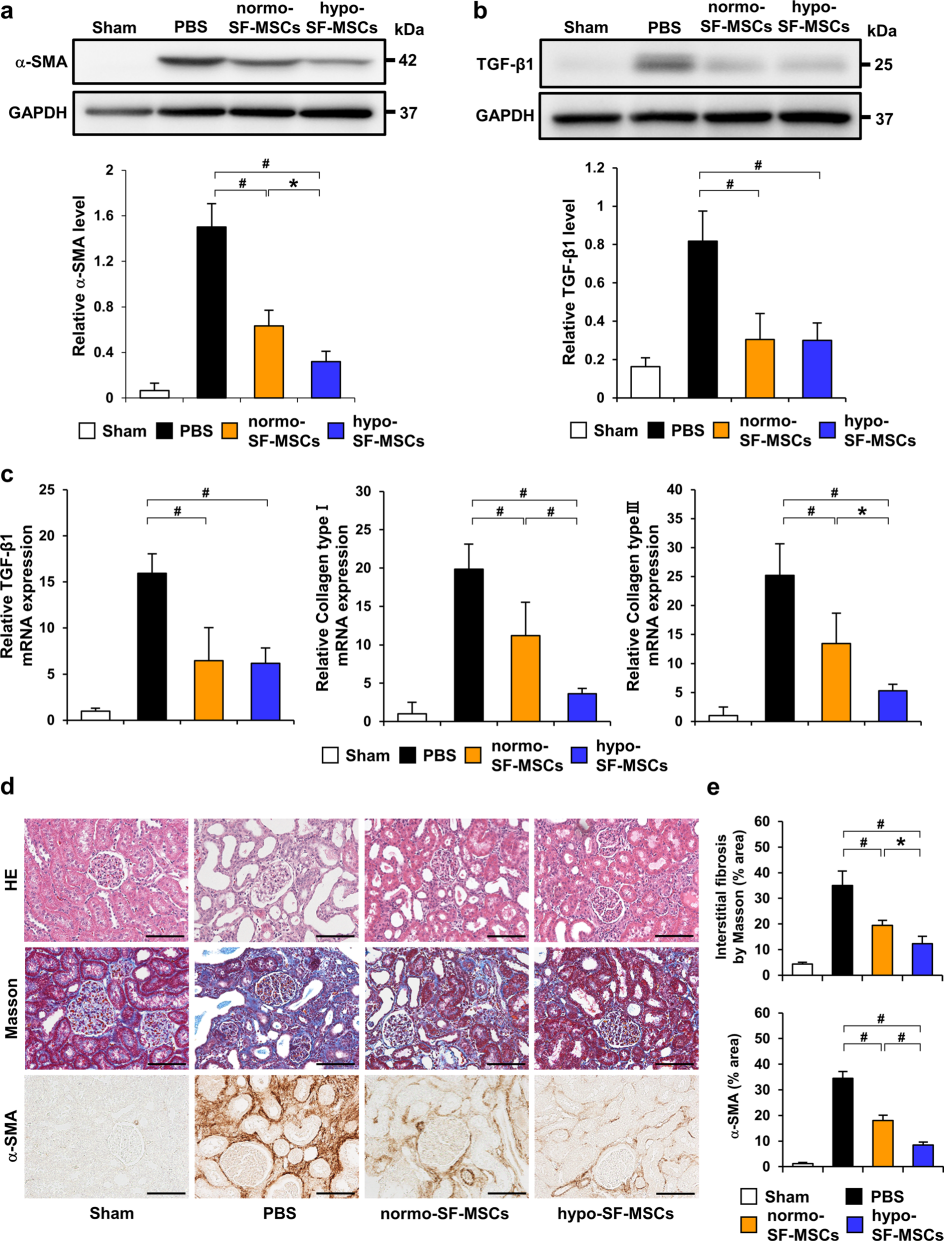
Hypo-SF-MSCs attenuate renal fibrosis more strongly than normo-SF-MSCs in IRI rats. **a, b** Western blot analysis of α-SMA and TGF-β1 in the kidney cortex of IRI rats at day 21 post-IRI. Graphs show densitometric analyses of α-SMA and TGF-β1 expression levels normalized to the GAPDH expression level (*n* = 5 in each group). **c** Expression levels of TGF-β1 and collagen type I and III mRNAs in the kidney cortex of IRI rats at day 21 post-IRI (*n* = 5 in each group). **d** Representative images of immunohistochemical staining of α-SMA as well as HE and Masson trichrome staining of kidney sections at day 21 post-IRI (scale bar = 100 µm). **e** Quantification of the interstitial fibrosis area and α-SMA-positive area as percentages of the total area (*n* = 5 in each group). Data are means ± S.D. ^#^*P* < 0.01, *P < 0.05 (one-way ANOVA followed by Bonferroni’s post-hoc test)

To clarify the effect of hypoxia-preconditioned SF-MSCs on renal fibrosis, we examined the expression of α-SMA and TGF-β1 in the IRI model that had been injected with PBS, normo-SF-MSCs, or hypo-SF-MSCs at 21 days post-IRI. As shown in Fig. 2a, the protein level of α-SMA was remarkably increased in IRI rats injected with PBS (PBS group). This increase was suppressed in IRI rats injected with normo-SF-MSCs (normo-SF-MSCs group) and further suppression was observed in those injected with hypo-SF-MSCs (hypo-SF-MSCs group) (Fig. 2a). In contrast, upregulation of the protein and mRNA levels of TGF-β1 in the IRI model was reduced by MSC treatments with no significant difference between normo-SF-MSCs and hypo-SF-MSCs groups (Fig. 2b, c). However, collagen type I and III mRNA levels were more reduced in the hypo-SF-MSCs group than in the normo-SF-MSCs group (Fig. 2c). Moreover, HE staining showed tubular dilatation and tubular cast formation in the PBS group and these tubulointerstitial injuries were suppressed by MSC treatments, particularly in the hypo-SF-MSCs group (Fig. 2d). Masson trichrome staining revealed that the area of interstitial fibrosis was more significantly reduced in the hypo-SF-MSCs group than in the normo-SF-MSCs group (Fig. 2d, e). Similarly, immunostaining revealed that the α-SMA-positive area was reduced in the hypo-SF-MSCs group compared with the normo-SF-MSCs group (Fig. 2d, e).

### Hypoxia-preconditioned SF-MSCs suppress infiltration of inflammatory cells in IRI rats

**Fig. 3:**
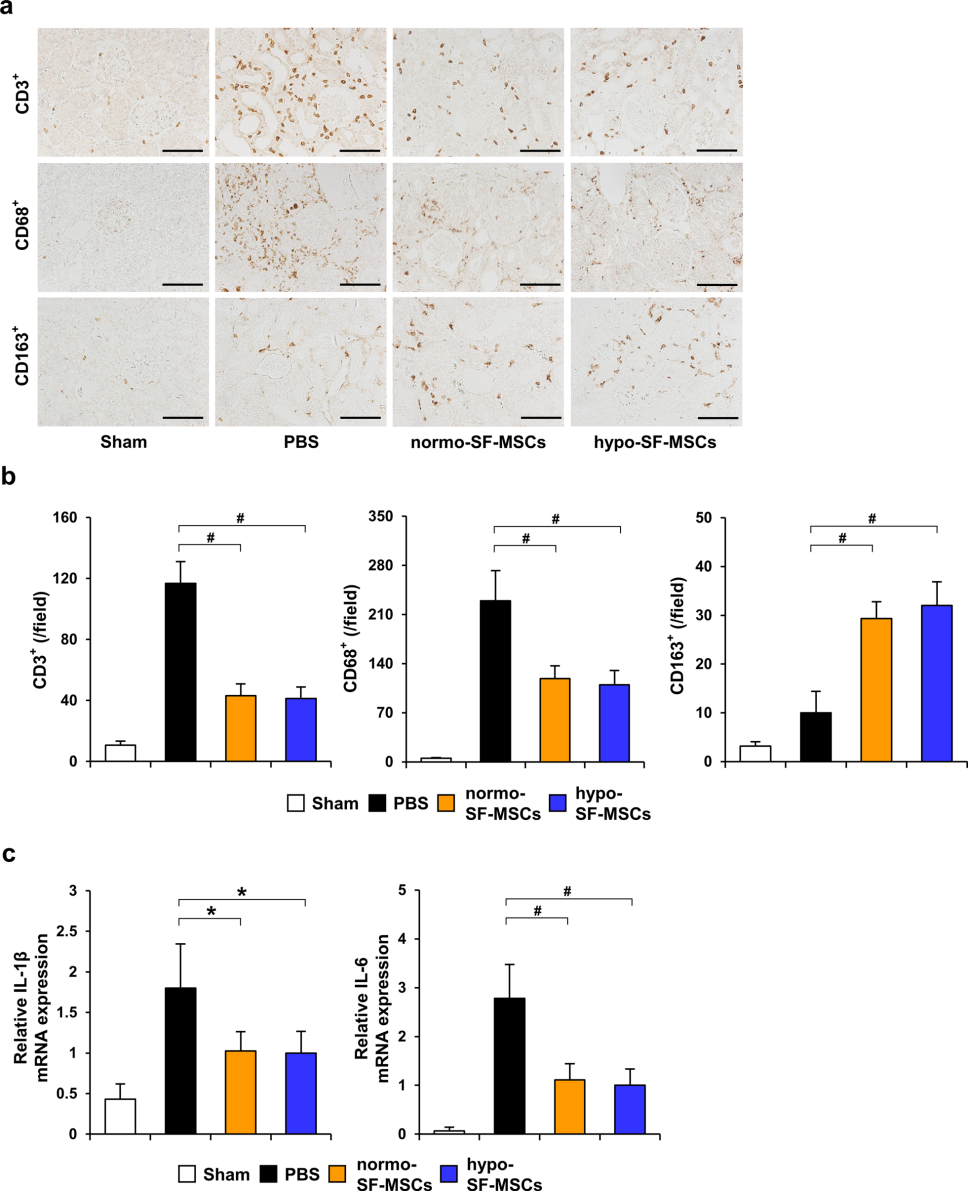
Hypo-SF-MSCs suppress infiltration of inflammatory cells to the same extent as normo-SF-MSCs in IRI rats. **a** Representative images of immunohistochemical staining of CD3, CD68, and CD163 in kidney sections at day 7 post-IRI (scale bar = 100 µm). **b** Quantification of CD3-, CD68-, and CD163-positive cells (*n* = 5 in each group). **c** Expression levels of IL-1β and IL-6 mRNA in the kidney cortex of IRI rats at day 7 post-IRI (*n* = 5 in each group). Data are means ± S.D. ^#^*P* < 0.01, *P < 0.05 (one-way ANOVA followed by Bonferroni’s post-hoc test)

Several studies have reported that persistence of inflammation leads to progression of fibrosis [30, 31]. Therefore, we next analyzed the expression of CD3 (T cell marker), CD68 (M1 and M2 macrophage marker), and CD163 (M2 macrophage marker) at 7 days post-IRI to investigate the anti-inflammatory effect of hypoxia-preconditioned SF-MSCs. As shown in Fig. 3a and b, immunohistochemical staining revealed that the number of CD3- and CD68-positive cells was increased in the PBS group. The infiltration of these cells was inhibited by MSC treatments with no significant difference between normo-SF-MSCs and hypo-SF-MSCs groups (Fig. 3a, b). Conversely, the number of cells positive for CD163, an immunosuppressive macrophage marker, was upregulated in both SF-MSCs groups with no significant difference between normo-SF-MSCs and hypo-SF-MSCs groups (Fig. 3a, b). We also found that IL-1β and IL-6 mRNA levels were upregulated in the PBS group, and these increases were suppressed in both normo-SF-MSCs and hypo-SF-MSCs groups with no significant difference between them (Fig. 3c).

### Hypoxic preconditioning does not affect the SF-MSC-induced change in the phenotype of macrophages from M1 to M2

**Fig. 4:**
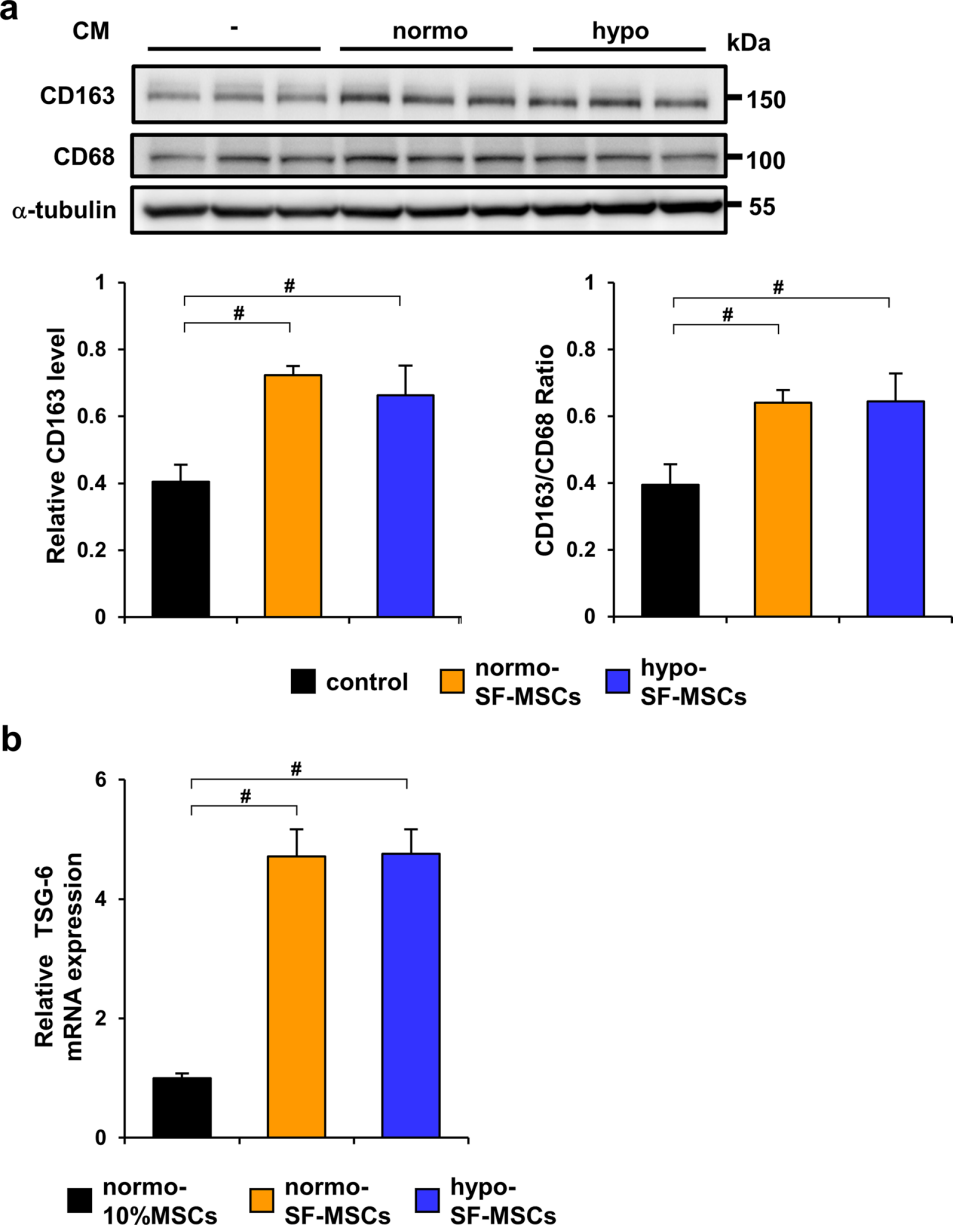
Hypoxic preconditioning does not affect SF-MSC-induced changes in the macrophage phenotype from M1 to M2. **a** Western blot analysis of CD163 and CD68 in THP-1 macrophages incubated with conditioned medium from normo-SF-MSCs or hypo-SF-MSCs. Graph shows densitometric analysis of the CD163 expression level normalized to CD68 and GAPDH expression levels (*n* = 5 in each group). **b** Expression level of TSG-6 mRNA in normo-10%MSCs, normo-SF-MSCs, and hypo-SF-MSCs (*n* = 5 in each group). Data are means ± S.D. ^#^*P* < 0.01 (one-way ANOVA followed by Bonferroni’s post-hoc test)

We have previously demonstrated that SF-MSCs change the phenotype of macrophages from proinflammatory M1 to immunosuppressive M2 compared with 10%MSCs [25]. Therefore, we investigated whether hypo-SF-MSCs further induced this change compared with normo-SF-MSCs. Although the protein level of CD163 was increased by both treatments with SF-MSC-CM, there was no significant difference between normo-SF-MSC-CM and hypo-SF-MSC-CM (Fig. 4a). Additionally, we found that the TSG-6 mRNA level in SF-MSCs was higher than that in normo-10%MSCs, but no significant difference was observed between normoxic and hypoxic conditions (Fig. 4b).

### Conditioned medium from hypoxia-preconditioned SF-MSCs strongly suppresses fibrotic changes by inhibition of TGF-β/Smad signaling

**Fig. 5:**
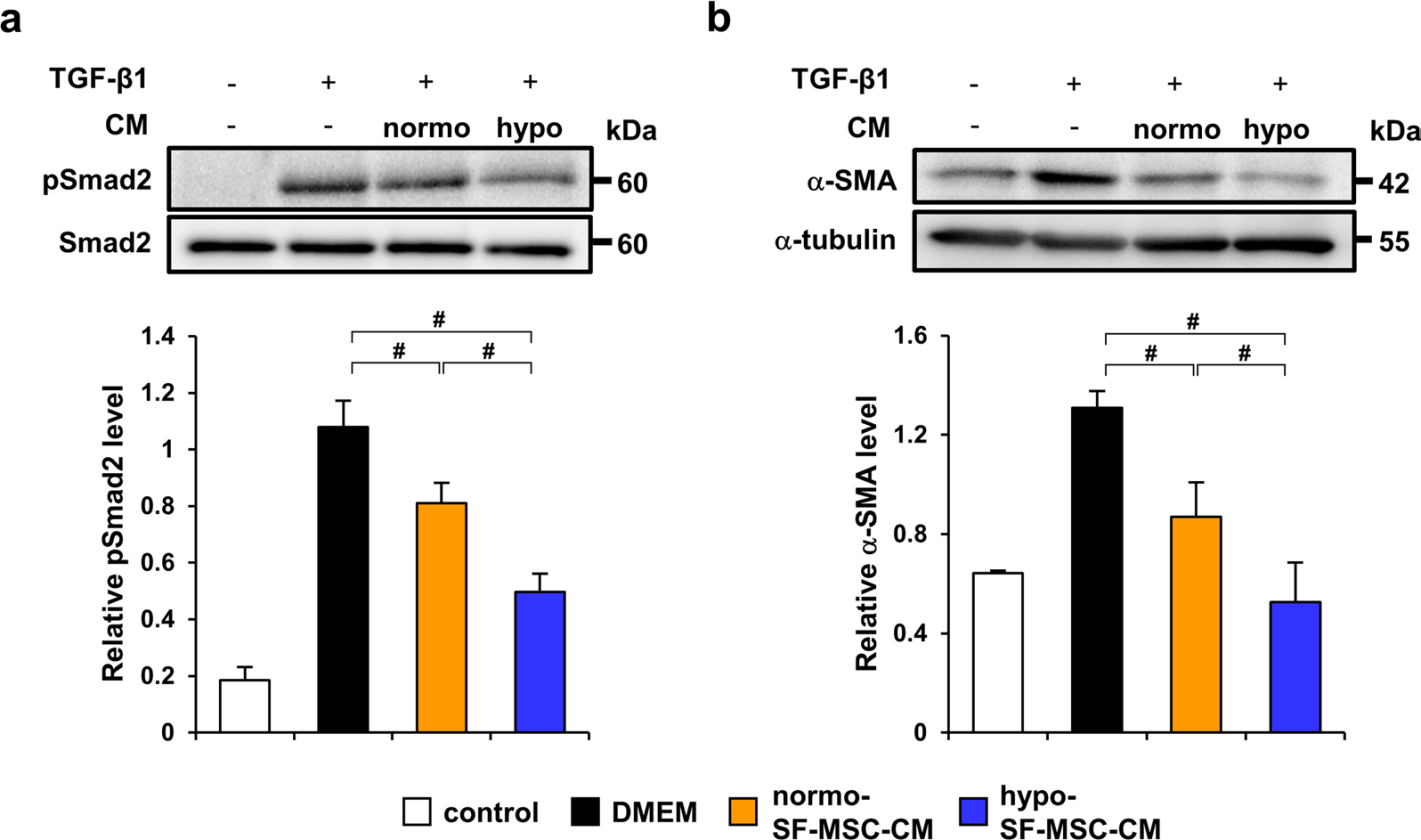
Conditioned medium from hypo-SF-MSCs strongly suppresses TGF-β1-induced fibrotic changes in HK-2 cells. **a** Western blot analysis of p-Smad2 in HK-2 cells stimulated with TGF-β1 for 30 min. Graph shows densitometric analysis of the p-Smad2 expression level normalized to the Smad2 expression level (*n* = 5 in each group). **b** Western blot analysis of α-SMA in HK-2 cells stimulated with TGF-β1 for 24 h. Graph shows densitometric analysis of the α-SMA expression level normalized to the α-tubulin expression level (*n* = 5 in each group). Data are means ± S.D. ^#^*P* < 0.01 (one-way ANOVA followed by Bonferroni’s post-hoc test)

We found that hypoxic preconditioning did not promote the anti-inflammatory effect of SF-MSCs despite the fact that it enhanced the anti-fibrotic effect. Therefore, we investigated whether hypo-SF-MSCs enhanced inhibition of TGF-β/Smad signaling using conditioned medium. To elucidate the direct anti-fibrotic effect of hypo-SF-MSCs on TGF-β/Smad signaling, we measured the protein levels of p-Smad2 and α-SMA in TGF-β1-stimulated HK-2 cells. We prepared normo-SF-MSC-CM and hypo-SF-MSC-CM and then stimulated HK-2 cells with TGF-β1 with or without each conditioned medium. The protein level of p-Smad2 was upregulated by TGF-β1 stimulation. This upregulation was reduced by treatment with normo-SF-MSC-CM and a further reduction was found after treatment with hypo-SF-MSC-CM (Fig. 5a). Similar results were observed for α-SMA protein expression (Fig. 5b).

### Hypoxic preconditioning enhances VEGF and HGF secretion from SF-MSCs

**Fig. 6:**
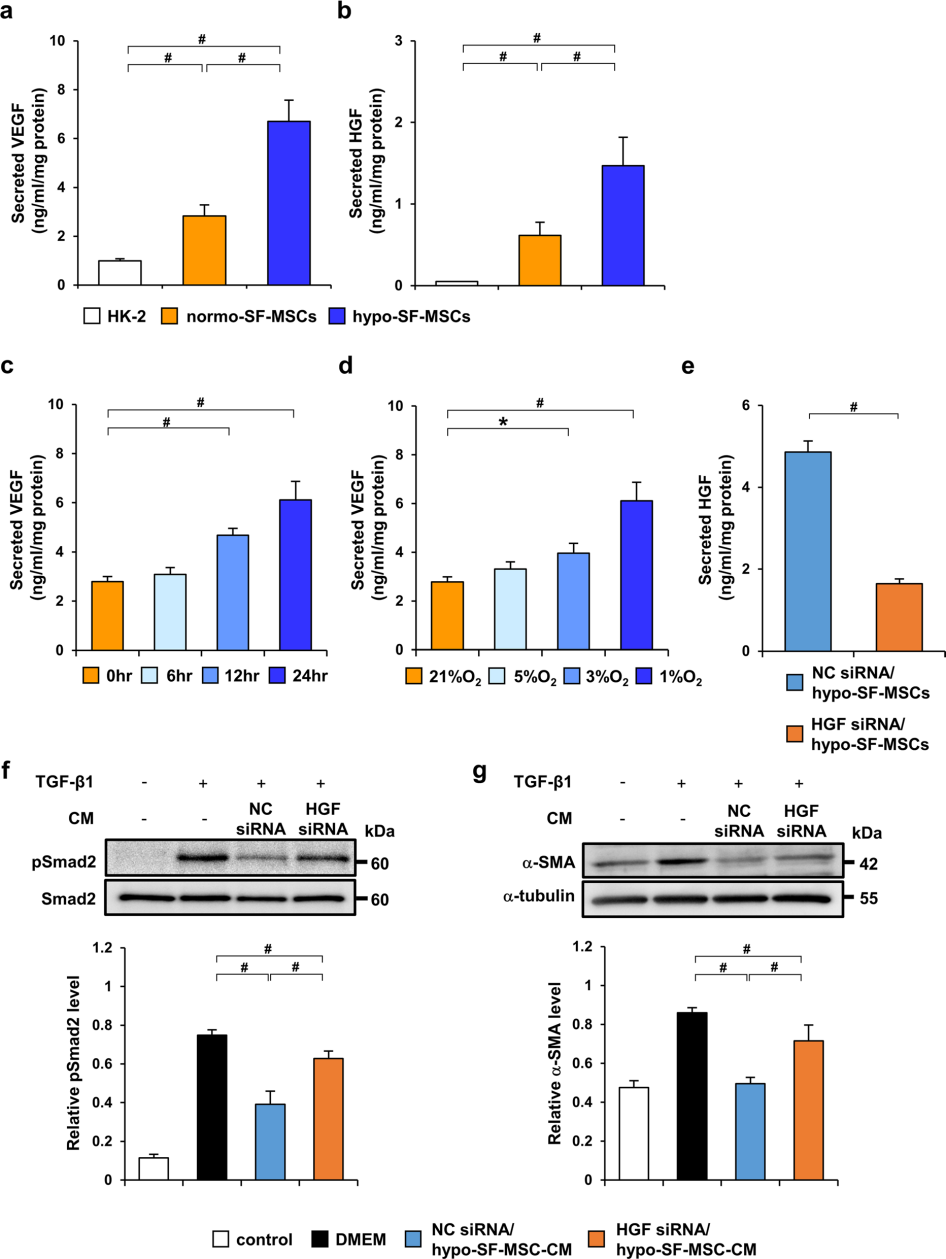
Knockdown of HGF in hypo-SF-MSCs reduces the anti-fibrotic effect in TGF-β1-stimulated HK-2 cells. The concentrations of vascular endothelial growth factor (VEGF) (**a**) and hepatocyte growth factor (HGF) (**b**) in each conditioned medium were measured by ELISAs (*n* = 5 in each group). **c** ELISA analysis showing the VEGF concentration in conditioned medium from SF-MSCs under hypoxia for various durations (*n* = 5 in each group). **d** ELISA analysis showing the VEGF concentration in conditioned medium from SF-MSCs cultured exposed to various O_2_ concentrations (*n* = 5 in each group). Hypo-SF-MSCs were transfected with HGF siRNA (HGF siRNA/hypo-SF-MSCs) or negative control siRNA (NC siRNA/hypo-SF-MSCs). **e** The HGF concentration was measured in conditioned medium from NC siRNA/hypo-SF-MSCs and HGF siRNA/hypo-SF-MSCs using an ELISA (*n* = 5 in each group). **f** Western blot analysis of p-Smad2 in HK-2 cells stimulated with TGF-β1 for 30 min. Graph shows densitometric analysis of the p-Smad2 expression level normalized to the Smad2 expression level (*n* = 5 in each group). **g** Western blot analysis of α-SMA in HK-2 cells stimulated with TGF-β1 for 24 h. Graph shows densitometric analysis of the α-SMA expression level normalized to the α-tubulin expression level (*n* = 5 in each group). Data are means ± S.D. ^#^*P* < 0.01, *P < 0.05 (one-way ANOVA followed by Bonferroni’s post-hoc test or Student’s t-test)

We previously found that hypoxic preconditioning has increased secretion of several renoprotective humoral factors, including VEGF and HGF [20]. To investigate whether hypoxic preconditioning enhanced secretion of these factors even under serum-free conditions, we measured the concentrations of VEGF and HGF in hypo-SF-MSC-CM and normo-SF-MSC-CM by ELISAs. As shown in Fig. 6a and b, we found upregulation of VEGF and HGF in hypo-SF-MSC-CM compared with normo-SF-MSC-CM. Next, to investigate the relationship between the duration of exposure to hypoxic conditions and VEGF secretion, SF-MSCs were incubated under 1% O_2_ conditions for 6, 12, or 24 h. ELISA analysis showed that the concentration of VEGF was increased over time for 24 h (Fig. 6c). Additionally, we cultured SF-MSCs with various O_2_ concentrations for 24 h. As shown in Fig. 6d, the concentration of VEGF was increased as the O_2_ concentration was decreased.

### Knockdown of HGF in hypoxia-preconditioned SF-MSCs reduces the anti-fibrotic effect in TGF-β1-stimulated HK-2 cells

We found that the HGF concentration in hypo-SF-MSC-CM was higher than that in normo-SF-MSC-CM. Hence, to identify the effect of HGF secreted from MSCs on TGF-β/Smad signaling, we prepared conditioned medium from hypo-SF-MSCs transfected with HGF siRNA (HGF siRNA/hypo-SF-MSC-CM) and negative control siRNA (NC siRNA/hypo-SF-MSC-CM). First, we confirmed successful knockdown of HGF in siRNA-transfected hypo-SF-MSCs by analyzing their conditioned medium (Fig. 6e). Moreover, to identify the effect of HGF secreted from MSCs on TGF-β/Smad signaling, we examined the effect of NC siRNA/hypo-SF-MSC-CM and HGF siRNA/hypo-SF-MSC-CM on p-Smad2 and α-SMA expression levels in TGF-β1-stimulated HK-2 cells. As shown in Fig. 6f, TGF-β1-induced expression of p-Smad2 was suppressed by treatment with NC siRNA/hypo-SF-MSC-CM. However, this inhibitory effect was weakened by treatment with HGF siRNA/hypo-SF-MSC-CM (Fig. 6f). Similar results were observed for α-SMA protein expression (Fig. 6g).

## Discussion

We have shown that serum-free medium attenuates senescence of MSCs, promoting their proliferative capacity and that hypoxia preconditioning further enhances the proliferative capacity of SF-MSCs. Additionally, administration of hypo-SF-MSCs strongly ameliorated renal fibrosis in comparison with that of normo-SF-MSCs in IRI rats, whereas the anti-inflammatory effect was almost equal for normo-SF-MSCs and hypo-SF-MSCs. In vitro experiments revealed that hypo-SF-MSC-CM more significantly inhibited TGF-β/Smad signaling than normo-SF-MSC-CM. Moreover, we found that hypoxic preconditioning increased HGF secretion, whereas knockdown of HGF in hypo-SF-MSCs attenuated the inhibition of TGF-β/Smad signaling. These findings suggest that serum-free conditions and hypoxic preconditioning synergistically enhance the anti-fibrotic effects of MSCs.

The persistence of inflammatory cell infiltration induces excessive production of extracellular matrix and fibrotic changes [30, 31]. The TGF-β/Smad signaling pathway is considered to play a central role in this process [32, 33]. In this study, although hypo-SF-MSCs and normo-SF-MSCs equivalently suppressed the infiltration of inflammatory cells and TGF-β1 expression, hypo-SF-MSCs strongly inhibited renal fibrosis compared with normo-SF-MSCs. These results are attributed to the fact that hypo-SF-MSCs more strongly suppressed TGF-β/Smad signaling, but not inflammation and TGF-β1 itself, compared with normo-SF-MSCs. We have previously demonstrated that hypoxia-preconditioned 10%MSCs enhance not only an immunosuppressive ability, but also a direct anti-fibrotic effect through inhibition of TGF-β/Smad signaling [20]. Interestingly, this study indicated that hypoxic preconditioning only enhanced the direct anti-fibrotic effect of SF-MSCs, but not the anti-inflammatory effect. This is probably because SF-MSCs had already exerted the strong anti-inflammatory effect through upregulation of TSG-6 as well as induction of polarization from M1 to M2 phenotype macrophages. Taken together, administration of hypo-SF-MSCs dramatically ameliorated renal fibrosis compared with that of normo-SF-MSCs by enhanced inhibition of the TGF-β/Smad signaling pathway.

The therapeutic capacity of MSCs is considered to depend on their ability to secrete beneficial factors to repair the injured tissue [34, 35]. We have previously reported that hypoxic preconditioning promotes VEGF and HGF secretion from 10%MSCs [20]. In this study, hypoxic preconditioning enhanced secretion of these factors even under serum-free conditions. Moreover, TSG-6 expression was increased in MSCs under serum-free conditions and this increase was sustained even under hypoxic conditions. These results suggest that serum-free conditions and hypoxic preconditioning do not antagonize each other, but enhance paracrine activity. Additionally, we found that knockdown of HGF in hypo-SF-MSCs reduced the anti-fibrotic effect on TGF-β1-induced fibrotic changes in HK-2 cells. Previous studies have shown that HGF antagonizes TGF-β/Smad signaling [36, 37]. Therefore, our data suggest that hypoxic preconditioning promotes HGF secretion and thereby enhances inhibition of TGF-β/Smad signaling, which leads to the pronounced anti-fibrotic effect of SF-MSCs.

For clinical application of MSCs, ex vivo culture is required to obtain a sufficient number of MSCs for treatments. Thus, the cell proliferative ability is important to obtain a large number of cells. In this study, we demonstrated that serum-free medium containing growth factors ameliorated cell senescence and promoted the cell proliferative ability and that hypoxic preconditioning further enhanced the proliferative ability of SF-MSCs. Therefore, also in terms of cell proliferative ability, preconditioning of SF-MSCs with hypoxia is a useful strategy. Sirt1 overexpression alleviates cellular senescence of MSCs and increases proliferative capacity [29]. However, genetic modification of MSCs requires complicated processes and cannot be performed easily. Therefore, hypo-SF-MSCs are particularly suitable for ex vivo culture.

This study has a limitation, namely the absence of experimental data on renal functions. A previous study has shown that a decrease in renal fibrosis alone is not sufficient to improve renal functions [38]. Blood biochemistry is often assessed to evaluate renal functions after IRI. However, in our animal model, the unilateral renal pedicle was clamped without contralateral nephrectomy. Therefore, blood biochemistry could not be used to evaluate renal functions of the injured side after IRI.

## Conclusions

In summary, administration of hypo-SF-MSCs further ameliorates renal fibrosis in IRI rats compared with normo-SF-MSCs with evidence that hypoxic preconditioning enhances the ability of SF-MSCs to inhibit the TGF-β/Smad signaling pathway. We also revealed that the combination of serum-free conditions and hypoxic preconditioning enhances paracrine activity without competing with each other and that HGF plays a pivotal role in hypo-SF-MSC-mediated inhibition of TGF-β/Smad signaling. Our results indicate that administration of hypoxia-preconditioned SF-MSCs has the potential to be a useful therapy to prevent the progression of renal fibrosis.

## Supplementary Information

**Additional file 1.** Characterization of MSCs.** a** Flow cytometry showing expression of surface markers on normo-10%MSCs. **b**, **c** Representative images of normo-10%MSCs after staining with Oil Red O and Alizarin Red S (scale bar = 100 μm).

## Data Availability

The data that support the findings of this study are available from the corresponding author upon reasonable request.

## Abbreviations

AKI: Acute kidney injury
CKD: Chronic kidney disease
DMEM: Dulbecco’s modified Eagle’s medium
ELISA: Enzyme-linked immunosorbent assay
FBS: Fetal bovine serum
HE: Hematoxylin and eosin
HGF: Hepatocyte growth factor
IL: Interleukin
IRI: Ischemia–reperfusion injury
MSCs: Mesenchymal stem cells
10%MSCs: MSCs cultured in DMEM containing 10% FBS
normo-10%MSCs: 10%MSCs cultured under 21% O2 conditions
SF-MSCs: MSCs cultured in serum-free medium
hypo-SF-MSCs: SF-MSCs cultured under 1% O2 conditions
normo-SF-MSCs: SF-MSCs cultured under 21% O2 conditions
hypo-SF-MSC-CM: Conditioned medium from hypo-SF-MSCs
normo-SF-MSC-CM: Conditioned medium from normo-SF-MSCs
HGF siRNA/hypo-SF-MSC-CM: Conditioned medium from hypo-SF-MSCs transfected with HGF siRNA
NC siRNA/hypo-SF-MSC-CM: Conditioned medium from hypo-SF-MSCs transfected with negative control siRNA
PBS group: IRI rats injected with PBS
hypo-SF-MSCs group: IRI rats injected with hypo-SF-MSCs
normo-SF-MSCs group: IRI rats injected with normo-SF-MSCs
p-Smad2: Phosphorylated Smad2
TSG-6: Tumor necrosis factor-α-induced protein 6
TGF: Transforming growth factor
VEGF: Vascular endothelial growth factor
WST: Water-soluble tetrazolium salts
