# Supplementary figures and images for: Serum-free medium and hypoxic preconditioning synergistically enhance the therapeutic effects of mesenchymal stem cells on experimental renal fibrosis
Source: Stem Cell Res Ther. 2021 Aug 23;12:472. doi: 10.1186/s13287-021-02548-7 (PMC8381539; doi:10.1186/s13287-021-02548-7)

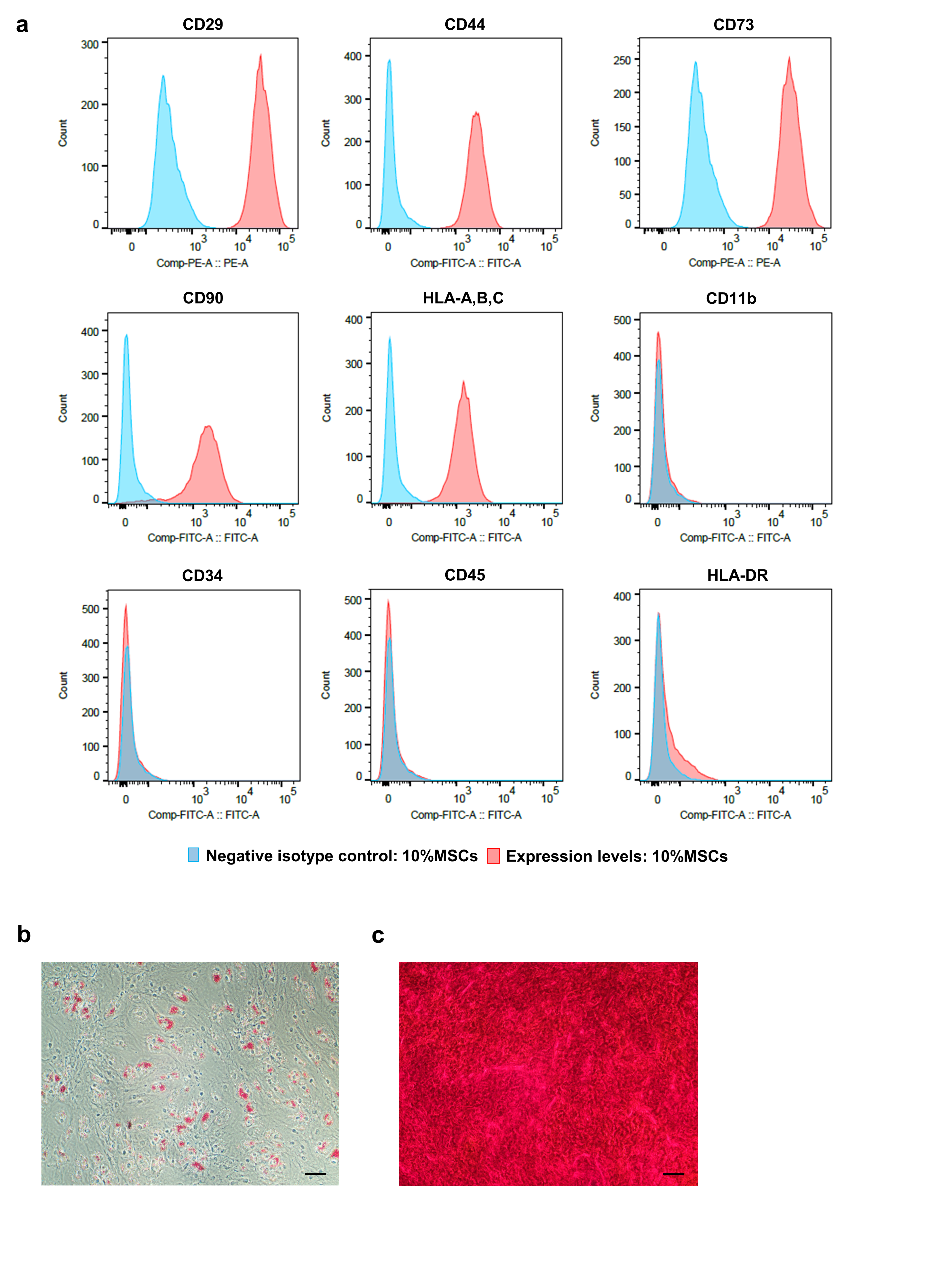

Supplement: Supplementary file 1 — Additional file 1. Characterization of MSCs. a Flow cytometry showing expression of surface markers on normo-10%MSCs. b, c Representative images of normo-10%MSCs after staining with Oil Red O and Alizarin Red S (scale bar = 100 μm). [file 13287_2021_2548_MOESM1_ESM.tif]
